# Atmospheric Hypoxia Limits Selection for Large Body Size in Insects

**DOI:** 10.1371/journal.pone.0003876

**Published:** 2009-01-07

**Authors:** C. Jaco Klok, Jon F. Harrison

**Affiliations:** School of Life Sciences, Arizona State University, Tempe, Arizona, United States of America; Universidade de Brasília, Brazil

## Abstract

**Background:**

The correlations between Phanerozoic atmospheric oxygen fluctuations and insect body size suggest that higher oxygen levels facilitate the evolution of larger size in insects.

**Methods and Principal Findings:**

Testing this hypothesis we selected *Drosophila melanogaster* for large size in three oxygen atmospheric partial pressures (aPO_2_). Fly body sizes increased by 15% during 11 generations of size selection in 21 and 40 kPa aPO_2_. However, in 10 kPa aPO_2_, sizes were strongly reduced. Beginning at the 12^th^ generation, flies were returned to normoxia. All flies had similar, enlarged sizes relative to the starting populations, demonstrating that selection for large size had functionally equivalent genetic effects on size that were independent of aPO_2_.

**Significance:**

Hypoxia provided a physical constraint on body size even in a tiny insect strongly selected for larger mass, supporting the hypothesis that Triassic hypoxia may have contributed to a reduction in insect size.

## Introduction

Recent geological models indicate a marked increase in atmospheric oxygen partial pressure (aPO_2_) to 32 kPa in the Permo-Carboniferous (≈300 million years ago), subsequently falling to 13 kPa in the Triassic [1]. These atmospheric oxygen partial pressure (aPO_2_) changes have been hypothesized to cause multiple major evolutionary events [2] including the appearance and subsequent extinction of giant insects and other taxa [3], [4]. Patterns of increasing tracheal investment in larger insects support this hypothesis [5], as do observations of positive relationships between aPO_2_ and body size in single- or multi-generational experiments with *Drosophila melanogaster* and other insects [6]. Large species likely result from many generations of selection for large body size driven by predation, competition or sexual selection [7]. Thus a crucial question is whether aPO_2_ influences the capacity of such selection to increase insect size. We tested that possibility by subjecting *Drosophila melanogaster* populations to truncation selection for large size for 11 generations in hypoxic (10 kPa), normoxic (21 kPa) and hyperoxic (40 kPa) aPO_2_, followed by three generations of normoxia without size selection.

Limited multigenerational studies with *Drosophila melanogaster* suggest that these insects might evolve larger body sizes when aPO_2_ is higher [8], [9]. However, body size can be affected by many factors, and it is not clear that interactions between oxygen and body size in the lab would occur in a similar manner in the field. *Drosophila melanogaster* exhibits strong changes in body size in response to artificial truncation selection for large size [10], and provide a convenient model for testing whether aPO_2_ influences the response of a species to strong selection for larger body size.

## Results

**Figure 1:**
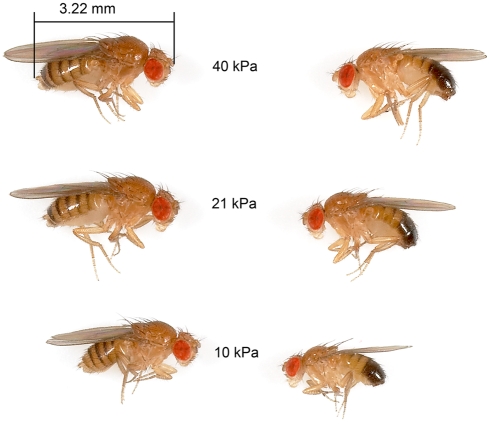
*Drosophila melanogaster* specimens (females left, males right) from the large size-selected populations maintained in their test aPO_2_s. The flies in 21 and 40 kPa had very similar body sizes but those maintained in 10 kPa exhibited strong size suppression despite having undergone strong size selection for 11 generations.

**Figure 2:**
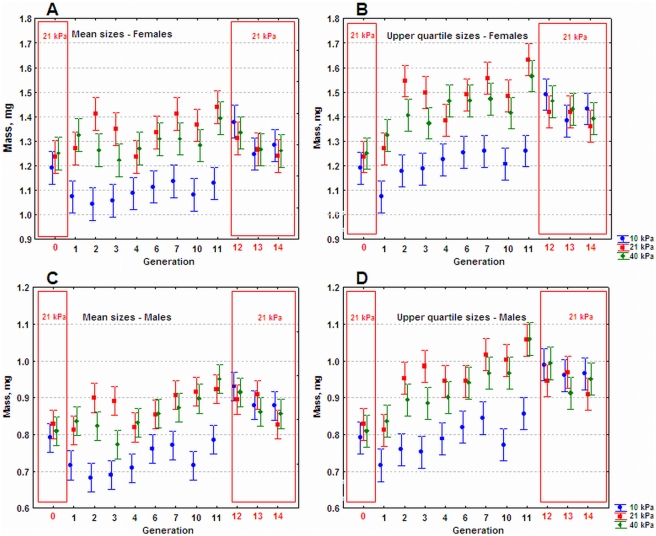
Plots of mass changes across generations. Mean adult masses (females above, males below) of five selected populations of *Drosophila melanogaster* (left), and mean masses of the largest quartile of those populations (values shown are the means±0.95 confidence intervals of the five population means for each treatment). Generation zero represents initial values of starting populations all reared in 21 kPa (included in red box). From generations 1–11, directional selection for large size was applied in either hypoxic (10 kPa, blue dots), normoxic (21 kPa, red squares) or hyperoxic (40 kPa, green diamonds) conditions. During generations 12–14, populations were returned to 21 kPa (included in red box) and no selection was performed. Non-overlapping 0.95 CI whiskers indicate significant differences. Due to questionable growth medium quality, generations 5, 8 and 9 were excluded from all analyses.

During size selection, we measured both mean population masses and also the masses of the largest quartile of flies, which were the flies selected to found generations 2 to 11. Both mean population masses and largest quartile masses of flies reared in 21 or 40 kPa aPO_2_ showed marked increases in response to size selection (Figs. 1, 2 and Table 1). After 11 generations, for the five populations of flies selected in 21 or 40 kPa aPO_2_, mean mass increased significantly by 11–17% over generation 0 values, and the upper quartile sizes increased by 25–32%. In most cases, there were no significant size differences between the 21 and 40 kPa groups (see Figs. 1, 2 and aPO_2_ effects in Table 1). By contrast, the flies selected for large size in 10 kPa aPO_2_ decreased in size during the initial selection generations, and then slowly increased (Fig. 1). After 11 generations of selection, the mean size of the five populations reared in 10 kPa aPO_2_ did not differ significantly from the starting populations (Fig. 2). Size selection significantly increased the upper quartile sizes of the flies reared in 10 kPa by 5–8% relative to the starting populations. Nevertheless, the sizes of all flies reared in 10 kPa aPO_2_ remained well below those of flies reared in 21 kPa or 40 kPa aPO_2_ throughout the selection period (see Figs. 1, 2 and aPO_2_ effects in Table 1).

**Table 1:** Statistical analyses of fly size variation at the start vs the end of positive size selection.

Effect	Population mean sizes			Upper quartile sizes		
	F	DF	p	F	DF	p
10 kPa vs 21 kPa: Generations 1 vs 11, during truncation selection for large size						
	Females			Females		
aPO_2_	69.09	2, 15	**<0.0001**	89.75	2, 15	**<0.0001**
Generation	95.98	2, 15	**<0.0001**	77.98	2, 15	**<0.0001**
aPO_2_×Generation	23.28	2, 15	**<0.0001**	24.07	2, 15	**<0.0001**
	Males			Males		
aPO_2_	45.32	2, 15	**<0.0001**	95.52	2, 15	**<0.0001**
Generation	39.52	2, 15	**<0.0001**	157.58	2, 15	**<0.0001**
aPO_2_×Generation	9.18	2, 15	**<0.0025**	14.18	2, 15	**<0.0004**
21 kPa vs 40 kPa: Generations 1 vs 11, during truncation selection for large size						
	Females			Females		
aPO_2_	0.05	2, 15	0.9531	4.36	2, 15	**<0.0322**
Generation	52.14	2, 15	**<0.0001**	36.20	2, 15	**<0.0001**
aPO_2_×Generation	3.04	2, 15	0.0781	1.52	2, 15	0.2500
	Males			Males		
aPO_2_	0.921	2, 15	0.4197	0.71	2, 15	0.5084
Generation	73.46	2, 15	**<0.0001**	62.90	2, 15	**<0.0001**
aPO_2_×Generation	7.23	2, 15	**<0.0063**	3.33	2, 15	0.0636

Repeated measures ANOVA statistics for the first and last generations that experienced directional selection for larger size, comparing hypoxic-reared (10 kPa, top) or hyperoxic-reared flies (40 kPa, bottom) to the control or normoxic-reared flies (21 kPa). Significant p values are boldfaced. In all cases, hypoxic-reared flies were significantly smaller than normoxic-reared flies, and responded differently than normoxic-reared flies. 10 kPa flies had a lesser increase in mass with size selection, indicated by significant aPO_2_×Generation terms. (F = F-ratio; DF = degrees of freedom).

When the populations were returned to normoxia (and random mating), the masses of the groups reared previously in the three different aPO_2_s converged within one generation toward the greater masses attained by the 21 and 40 kPa groups. Regardless of prior aPO_2_, the populations' mean increase in mass relative to generation 0 was 2–11%, while the largest quartile flies increased in size by 12–21% (Table 2). Clearly truncation selection successfully changed both the mean values and the size distributions of these populations. The similarity of the masses of the groups in generations 12–14 indicates that the selection-induced genetic changes related to size were similar and independent of historical aPO_2_ during selection.

**Table 2:** Statistical analyses of variation of initial fly sizes vs. the size of flies post-selection–all reared in normoxic conditions.

Effect	Population mean sizes			Upper quartile sizes		
	F	DF	p	F	DF	p
10 kPa vs 21 kPa: Generations 0 pre- vs 13 post-size selection						
	Females			Females		
aPO_2_	1.06	2, 15	0.3722	0.91	2, 15	0.4222
Generation	3.81	2, 15	**<0.0459**	20.58	2, 15	**<0.0001**
aPO_2_×Generation	0.17	2, 15	0.8430	0.52	2, 15	0.6062
	Males			Males		
aPO_2_	3.55	2, 15	0.0545	1.43	2, 15	0.2713
Generation	7.89	2, 15	**<0.0045**	24.29	2, 15	**<0.0001**
aPO_2_×Generation	0.02	2, 15	0.9778	0.20	2, 15	0.8252
21 kPa vs 40 kPa: Generations 0 pre- vs 13 post-size selection						
	Females			Females		
aPO_2_	0.31	2, 15	0.7354	1.42	2, 15	0.2715
Generation	1.38	2, 15	0.2826	24.82	2, 15	**<0.0001**
aPO_2_×Generation	0.52	2, 15	0.6037	0.16	2, 15	0.8570
	Males			Males		
aPO_2_	2.82	2, 15	0.0915	2.35	2, 15	0.1292
Generation	13.19	2, 15	**<0.0005**	35.46	2, 15	**<0.0001**
aPO_2_×Generation	10.89	2, 15	**<0.0012**	14.80	2, 15	**<0.0003**

Repeated Measures ANOVA statistics (α = 0.05) for the starting populations at Generation 0 vs the second generation (Generation 13) of populations post-size selection and returned to normoxia. Although all these flies were reared in normoxia, the analyses compare previously hypoxic-selected (10 kPa, top) or previously hyperoxic-selected flies (40 kPa, bottom) to control flies that experienced size selection in normoxia (21 kPa). Significant p values are boldfaced. In general, flies were larger in generation 13 than in the starting populations, indicating evolution of larger size in response to truncation selection (significant generation effects). However, there were no significant effects of the aPO_2_ during the period of size selection. (F = F-ratio; DF = degrees of freedom).

## Discussion

Our data did not support the hypothesis that atmospheric hyperoxia would enable the evolution of larger insects in a strong size selective environment, as hyperoxic rearing did not allow flies to reach larger sizes relative to normoxic rearing. In general, phenotypic plastic responses of *D. melanogaster* body size to 40 kPa aPO_2_ are relatively small (3–6%) [11] and it is not surprising that selection can overcome such a minor plastic effect. Conceivably, a different result would occur at a less extreme level of hyperoxia. Forty kPa aPO_2_ is near the highest level of oxygen for successful rearing of some *D. melanogaster* strains [12], and thus at this aPO_2_ there may be oxidative stress that counters positive effects of hyperoxia on size. However, it has also been demonstrated that insects can control their spiracular openings to limit the potentially detrimental effect of too much oxygen [13]. Additionally, with larger or different populations, and more variance available for selection, it is possible that hyperoxia might affect responses to selection. Also, one should take into account that *D. melanogaster* is a very small insect, and potentially the interactions between body size and oxygen delivery might differ in much larger insects, such as the giant Palaeozoic palaeopterans. The correlations between increased aPO_2_ during this era [1], [2] and insect gigantism [2]–[4], as well as experimental evidence of increased body size of insects reared in hyperoxia [6] lend support to the hypothesis that atmospheric hyperoxia contributed to the evolution of gigantism.

By contrast, this study's data convincingly show that hypoxia can limit the size of insects, even when they are strongly selected for large size (Fig. 1). We cannot exclude the possibility that with larger population sizes and more generations, that the hypoxic-reared flies could attain the size of flies selected in normoxia. However, the trends in our experiments suggest the alternative, that greater populations and time would increase the divergence induced by aPO_2_ (Fig. 2).

Is it reasonable to extrapolate from the small *D. melanogaster* to the giant insects of the Palaeozoic? Hypoxia suppresses size in most of the modern insects that have been studied, at least in single generation studies [6]. These plastic effects of hypoxia on size in *D. melanogaster* are possibly mediated via oxygen-dependent signalling pathways regulating growth and developmental processes such as the ISS pathway (Insulin/Insulin like growth factor signalling glucose transport and cell growth), IDGFs (chitinase related imaginal disc growth factors), ADGFD (adenosine-deaminase related growth factor) [14], HIF-1α (hypoxia inducible factor) [15], [16], or via Tuberous Sclerosus Complex 2 (Tsc2) or Redd1-mediated suppression of TOR signalling [17], [18]. Analogous representatives of these signalling pathways have been characterized in *Hydra* (Coelenterata) [19], *Caenorhabditis elegans* (Nematoda) [20], [21], *Daphnia magna* (Crustacea) [22], *D. melanogaster* (Insecta) [14], [22], various mammals [23], yeast and *Arabidopsis* [24]. This broad distribution of oxygen-dependent growth among organisms indicates that these signalling pathways originated in their common ancestry at least 500 million years ago [24], are highly conserved among eukaryotes, and therefore likely also regulated the development of the Palaeozoic giant insect species such as *Meganeura monyi* and *Meganeuropsis permiana* (Order Protodonata) [25] and *Mazothairos enormis* (Order Palaeodictyoptera) [26]. Thus, our data, demonstrating strong size suppression in a small insect selected for large size, strongly supports the hypothesis that decreased aPO_2_ could explain the giant palaeopteran species' extinction during the progressively hypoxic aPO_2_ across the Permo-Triassic boundary [1].

## Materials and Methods

To test this potential effect of atmospheric oxygen concentration on positive size selection, we performed truncation selection for 11 generations on five populations of *D. melanogaster* in 10, 21 and 40 kPa aPO_2_ respectively. To maximize genetic diversity, starting populations were derived by outbreeding five unrelated *Drosophila melanogaster* lines (Tucson *Drosophila* Stock Center numbers: 14021-0231.20, 14021-0231.24, 14021-0231.35, 14021-0231.38, 14021-0231.43). As a precaution to unpredictable events during selection, these outbred stocks were treated with tetracycline and rifampicin for 3–5 generations prior to the start of truncation selection procedures to eliminate *Wolbachia* infections [27], [28]. Two antibiotic-free generations preceded selection experiments, and the experimental media lacked antibiotics.

### Generation 0

We split our outbred stock into 15 populations (5 replicates per aPO_2_, each started with 30♀ and 20♂ newly eclosed flies, <48 hours old). The flies were cold-anaesthetized (1 hr at 4±1°C) [29], weighed individually (Mettler MX 5, ±0.001 mg), and placed in 237 ml bottles with 50 ml standard yeast-based *Drosophila* growth medium. The bottles were kept in an incubator (Percival, Boone IO, 25°C, 12L∶12D photoperiod) inside three air-tight chambers, each connected to a Sable Systems ROXY-8 paramagnetic oxygen regulation system that regulated aPO_2_ at 10, 21 and 40 kPa (www.sablesys.com/roxy8.html). Adult flies were allowed to mate randomly and oviposit for four days after which they were removed to limit larval densities to <250/bottle.

### Size selection–Generations 1 to 11

To determine mean population masses, we weighed 30♀ and 20♂ newly eclosed adult flies (haphazardly-chosen) per population. Of these, the largest 10♀ and 6♂ per population were placed in new bottles and served as a portion of the founders of the next generation. From the other flies, we visually selected and individually weighed the largest 35♀ and 25♂. Preliminary analyses confirmed that we could visually select flies whose average mass did not differ significantly from actual largest masses in each population, ANOVA: F_4, 45_ = 0.619, p = 0.65. These visually selected 35♀ and 25♂ were then weighed individually and sorted according to mass. From these, the largest 20♀ and 14♂ were added to the largest 10♀ and 6♂ mentioned above. This additional procedure ensured that we selected flies from the actual largest quartile of the population. Together these size-selected 30♀ and 20♂ adults founded the next generations.

### Return to normoxia

For generations 12–14, selection ceased and populations were reared at 21 kPa. Randomly selected adults (30♀ and 20♂) founded each generation, and we continued to measure mean and largest upper quartile masses as described above, because prior research suggests that the effects of oxygen may be stronger on maximum sizes compared to mean sizes [30], [31].

### Statistical analyses

Data sets for ‘mean population masses’ and ‘upper quartile masses’ were compiled and analyzed separately using STATISTICA 8 (www.StatSoft.com). Females and males were analyzed separately. At each generation, the mean masses of each sex for each population and the mean mass of the largest quartile of flies for each sex and population were used as data, giving an n = 5 for each selection group. A repeated measures ANOVA design tracked the changes in size across generations for each oxygen concentration.
